# ﻿*Petrocodonparviflorus* (Gesneriaceae), a new species identified by both morphological and molecular evidence from limestone caves in Guangxi, China

**DOI:** 10.3897/phytokeys.260.153384

**Published:** 2025-07-28

**Authors:** Rui-Ning Liu, Ke Tan, Ping-Ya Chen, Xin-Xiang Bai, Chi Xiong, Song-Tao He, Fang Wen

**Affiliations:** 1 Forestry College, Guizhou University, CN-550025 Guiyang, China Guangxi Institute of Botany, Guangxi Zhuang Autonomous Region and Chinese Academy of Sciences Guilin China; 2 Guangxi Key Laboratory of Plant Conservation and Restoration Ecology in Karst Terrain, Guangxi Institute of Botany, Guangxi Zhuang Autonomous Region and Chinese Academy of Sciences, CN-541006 Guilin, China Guizhou University Guiyang China; 3 Gesneriad Committee of China Wild Plant Conservation Association (GC), National Gesneriaceae Germplasm Resources Bank (NGGRB) of GXIB, Gesneriad Conservation Center of China (GCCC), Guilin Botanical Garden, Guangxi Zhuang Autonomous Region and Chinese Academy of Sciences, CN-541006 Guilin, China Guilin Botanical Garden, Guangxi Zhuang Autonomous Region and Chinese Academy of Sciences Guilin China; 4 Haikou Customs Technical Center, CN-570311 Haikou, China Haikou Customs Technical Center Haikou China

**Keywords:** ITS, limestone flora, molecular phylogeny, *Petrocodon*, taxonomy, *trn*L-F

## Abstract

*Petrocodonparviflorus*, a new species of Gesneriaceae, is a typically cave-dwelling species from the limestone region in Guangxi, China. Morphologically, this new species resembles *P.lui*, but can be readily distinguished by differences in corolla limb lobe shape, calyx lobe surface texture, pistil style length, and central staminode length. Molecular evidence supports its close phylogenetic relationship with *P.lui* despite their morphological distinctness. Based on the IUCN Red List Categories and Criteria, this species is assessed as Vulnerable (VU, D2).

## ﻿Introduction

*Petrocodon* Hance is predominantly distributed in limestone regions, with a minority of species occurring in Danxia landform areas, such as *P.asterocalyx* F.Wen, Y.G.Wei & R.L.Zhang (Zhang et al. 2018), *P.chishuiensis* Z.B.Xin, F.Wen & S.B.Zhou (Zhou et al. 2020), *P.wui* F.Wen & R.B.Zhang (Zhang et al. 2023) and *P.zhonglii* X.Z. Shi, J.X. Fu & Li H. Yang (Shi et al. 2025). Many species exhibit typical troglophytic adaptations, such as *P.tiandengensis* (Yan Liu & B.Pan) A.Weber & Mich.Möller, *P.curvitubus* J.X.Wei, B.Pan & T.Ding, *P.wenshanensis* Xin Hong, W.H.Qin & F.Wen (Weber et al. 2011; Li et al. 2020; Wei et al. 2025). Before the description of this species, the genus comprised 60 species and 1 variety (GRC 2025; POWO 2025; Shi et al. 2025; Tan et al. 2025).

During fieldwork in Fengshan County, Guangxi, in April 2019, we discovered an unknown gesneriad growing on moist, shady rock surfaces within a large karst cave. Based on its habitat and morphological characteristics, such as rosette plant, elliptic or ovate leaves, infundibuliform corolla, straight filaments, and capitate stigma, we provisionally identified that it belonged to *Petrocodons.l.* as originally identified. To confirm its taxonomic identity, we conducted detailed morphological comparisons and molecular phylogenetic analyses. These analyses collectively confirmed that the gesneriad from Fengshan County represents a new species within the redefined *Petrocodons.l.*, which is formally described here.

## ﻿Material and methods

### ﻿Morphological comparison

Dried herbarium specimens of the new species are deposited in IBK. Additionally, we took photographs documenting the habitat and plant habits during fieldwork in the wild. Morphological characters were measured using living and dried materials in the laboratory. We also compared the new species with digitized type specimens of related species from several herbaria, including PE (https://pe.ibcas.ac.cn/), GXMG (http://www.gxyyzwy.com/), IBK (http://www.cfh.ac.cn/subsite/Albums.aspx?siteid=IBK), and so on. The terminology used in the description follows Wang et al. (1998) and Harris and Harris (2001). The assessment of conservation status adheres to the guidelines outlined in the IUCN Red List Categories and Criteria (IUCN 2024).

### ﻿DNA extraction, PCR amplification and sequencing

To verify the taxonomic status of the new species within *Petrocodon*, we conducted a phylogenetic analysis using the two gene regions commonly used in the phylogenetic reconstruction of Gesneriaceae (ITS and *trn*L-F). Leaf materials for DNA extraction of the proposed new species were dried in a vascular bag with silica gel in the field and DNA was extracted using a modified CTAB protocol (Allen et al. 2006). The ITS and *trn*L-F were amplified using the ITS primers ITS1 and ITS4 (Wendel et al. 1995) and the *trn*L-F (Taberlet et al. 1991), respectively. Subsequently, we downloaded most of the ITS and *trn*L-F sequences of 35 *Petrocodon* species and 2 species from *Primulina* Hance as outgroups from NCBI GenBank. Sequences used in this study are listed in Table 1.

**Table 1.** List of *Petrocodonparviflorus* and 36 *Petrocodon* species used in the phylogenetic analysis, including respective Genbank accession and voucher numbers.

Species	*trn*L-*trn*F	ITS1/4	Voucher/Herbarium barcode
*Petrocodonainsliifolius* W.H. Chen & Y.M. Shui	MN637548	MN627898	LPW2013089 (KUN)
*Petrocodonalbinervius* D.X. Nong & Y.S. Huang	ON959495	ON950050	WF001 (IBK)
*Petrocodonasterocalyx* F.Wen, Y.G.Wei & R.L.Zhang	KC904957	KC904954	FW-2013 (IBK)
Petrocodon asterostriat*us* F. Wen, Y.G. Wei & W.C. Chou	ON959497	ON950052	- / IBK
*Petrocodonchishuiensis* Z.B. Xin, F. Wen & S.B. Zhou	KF680503	KF680504	FW-2014 (IBK)
*Petrocodonchongqingensis* F.Wen, B.Pan & L.Y.Su	MN637569	MN627918	LPW2018004 (IBK)
*Petrocodonconfertiflorus* H.Q.Li & Y.Q.Wang	MN637556	MN627906	LPW2018004 (SN)
*Petrocodoncoriaceifolius* (Y.G.Wei) Y.G.Wei & Mich.Möller	KY796060	KY796058	IBK:W.-B. Xu s.n. (IBK)
*Petrocodondealbatus* Hance	KR476565	KR337020	LJM1209291
*Petrocodondealbatus* var. denticulatus (W.T. Wang) W.T. Wang	JF697590	JF697578	KN03 (PE)
*Petrocodonferrugineus* Y.G. Wei	MN637550	MN627900	LPW2018013 (IBK)
*Petrocodonhechiensis* (Y.G.Wei, Yan Liu & F.Wen) Y.G.Wei & Mich. Möller	KR476563	KR337018	- / IBK
*Petrocodonhispidus* (W.T. Wang) A.Weber & Mich.Möller	MN637566	MN627915	LPW2012010 (PE)
*Petrocodonhunanensis* X.L. Yu & Ming Li	MN637570	MN627919	LPW2014014 (CSFI)
*Petrocodonintegrifolius* (D. Fang & L.Zeng) A.Weber & Mich.Möller	MN637571	MN627920	LPW2013040 (GXMI)
*Petrocodonionophyllus* F.Wen, S.Li & B.Pan	ON959496	ON950051	WF010 (IBK)
*Petrocodonjasminiflorus* (D.Fang & W.T.Wang) A.Weber & Mich.Möller	MN637568	MN627917	LMT2012002
*Petrocodonjiangxiensis* F.Wen, L.F.Fu & L.Y.Su	MH699398	MH699397	WF170502 (IBK)
*Petrocodonjingxiensis* (Yan Liu, H.S.Gao & W.B.Xu) A.Weber & Mich.Möller	MN637544	MN627894	LPW2014067 (IBK)
*Petrocodonlancifolius* F.Wen & Y.G.Wei	MN637552	MN627902	LPW2018031 (IBK)
*Petrocodonlaxicymosus* W.B.Xu & Yan Liu	MN637549	MN627899	LPW2018019 (IBK)
*Petrocodonlonggangensis* W.H.Wu & W.B.Xu	MN637546	MN627896	LPW2013038 (IBK)
*Petrocodonlongitubus* Cong R.Li & Yang Luo	MN637543	MN627893	Wangmo1073
*Petrocodonlui* (Yan Liu & W.B.Xu) A.Weber & Mich.Möller	HQ632938	HQ633035	Y.G.Wei 8012 (IBK)
*Petrocodonmultiflorus* F. Wen & Y.S. Jiang	KM232660	KJ475411	HJ01-2 (IBK)
*Petrocodonniveolanosus* (D.Fang & W.T.Wang) A.Weber & Mich.Möller	MN627922	MN637573	LPW2015051 (E)
*Petrocodonparviflorus* sp. nov.	PV468307	PV550299	WF012 (IBK)
*Petrocodonpseudocoriaceifolius* Yan Liu & W.B.Xu	MN637547	MN627897	LPW2018032 (IBK)
*Petrocodonpulchriflorus* Y.B. Lu & Q. Zhang	MN637565	MN627914	LPW2018018 (IBK)
*Petrocodonretroflexus* Q. Zhang & J. Guo	MN637540	MN627890	G43 (IBK)
*Petrocodonscopulorum* (Chun) Yin Z.Wang	MN637562	MN627911	LPW2018003
*Petrocodontiandengensis* (Yan Liu & B.Pan) A.Weber & Mich.Möller	JX506850	JX506960	09413 (IBK)
*Petrocodontongziensis* R.B.Zhang & F.Wen	MF872618	MF872617	Ren-Bo Zhang SBQ09383 (ZY)
*Petrocodonurceolatus* F.Wen, H.F.Cen & L.F.Fu	MN637559	MN627909	LPW2018029 (IBK)
*Petrocodonviridescens* W.H. Chen, Mich. Möller & Y.M. Shui	MN637572	MN636954	Li,P.W. & Liu,M.T. 85339 (KUN)
*Petrocodonwui* F.Wen & R.B.Zhang	OQ716553	OQ694978	WF065 (IBK)
*Primulinapinnata* (W.T.Wang) Yin Z.Wang	FJ501526	FJ501349	G26
*Primulinadryas* (Dunn) Mich.Möller & A.Weber	FJ501524	FJ501348	C7a

### ﻿Phylogenetic analysis

We aligned the newly obtained sequences together with sequences retrieved from NCBI GenBank using MAFFT version 7.463 (Katoh and Standley 2013), and further adjusted them manually in MEGA 11.0.13. Thereafter, we use ModelFinder (Kalyaanamoorthy et al. 2017) to determine the most suitable models for the subsequent Maximum Likelihood (ML) analysis and Bayesian Inference (BI). The result suggested K3Pu+F+R2 model and the HKY+F+I+G4 model were selected as the optimal substitution models for maximum likelihood (ML) analysis and Bayesian inference (BI), respectively. Bayesian Inference (BI) analysis was conducted using MrBayes version 3.2.7 (Ronquist et al. 2012), with two independent Markov Chain Carlo (MCMC) analyses were run for 10 million generations, and sampled every 1000 generations. The first 25% trees were discarded as burn-in, and the remaining trees were summarized in a 50% majority-rule consensus tree with the posterior probabilities (PP). Maximum Likelihood (ML) analyses were employed to reconstruct the phylogeny in IQ-TREE version 2.1.4 (Nguyen et al. 2015), with the optimal substitution models to carry out 1000 bootstrap (BS) replicates. Both MrBayes and IQ-TREE analyses were integrated into PhyloSuite version 1.2.3 (Zhang et al. 2020). The resultant tree was displayed using iTOL version 7 online software (Letunic and Bork 2021).

## ﻿Results

### ﻿The systematic position of the putative new species

**Figure 1.**
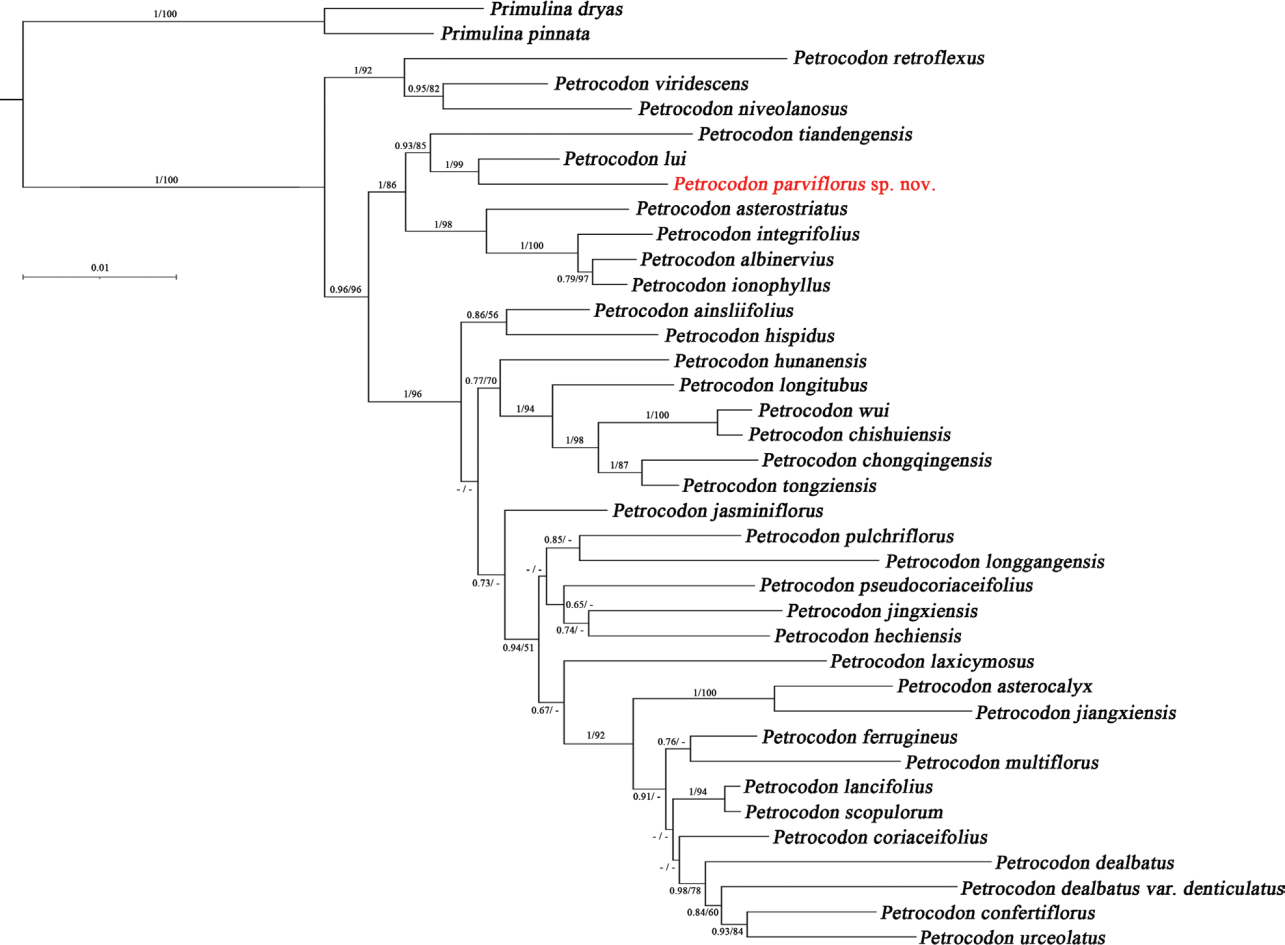
Phylogenetic tree of *Petrocodonparviflorus* taxa and 35 *Petrocodon* species, based on the combined chloroplast gene *trn*L-F and ITS data matrices. Posterior probability (PP) and Bootstrap value (BS) are showed above branches (only shown if BS > 50%). *Petrocodonparviflorus* in red.

The new species is strongly supported in a clade (BS = 85%, PP = 93%) (Fig. 1), which also includes *P.tiandengensis* (Yan Liu & B.Pan) A.Weber & Mich.Möller and *P.lui* (Yan Liu & W.B.Xu) A.Weber & Mich.Möller (Weber et al. 2011). Phylogenetically, the new species is sister with *P.lui* with high support (BS = 99%, PP = 100%). Moreover, the morphologies of these two relative species are obviously different.

### ﻿Taxonomic treatment

#### ﻿ Petrocodon parviflorus

Taxon classificationPlantaeLamialesGesneriaceae

F.Wen & K.Tan

7F8CDCF1-60CC-5532-887D-79BD5B56EFEE

Figs 2 , 3 , 4 , 5

##### Diagnosis.

The new species resembles *P.lui* in leaf blade shape, but can be easily distinguished from the latter by corolla limb lobes oval (*vs.* oblong, obovate to suborbicular), longer style length 10–11.5 mm long (*vs.* 4–6 mm long), longer central staminode 1–1.3 mm long (*vs.* 0.5–0.8 mm long), calyx lobes sparsely puberulent abaxially (*vs.* glabrous abaxially).

**Figure 2.**
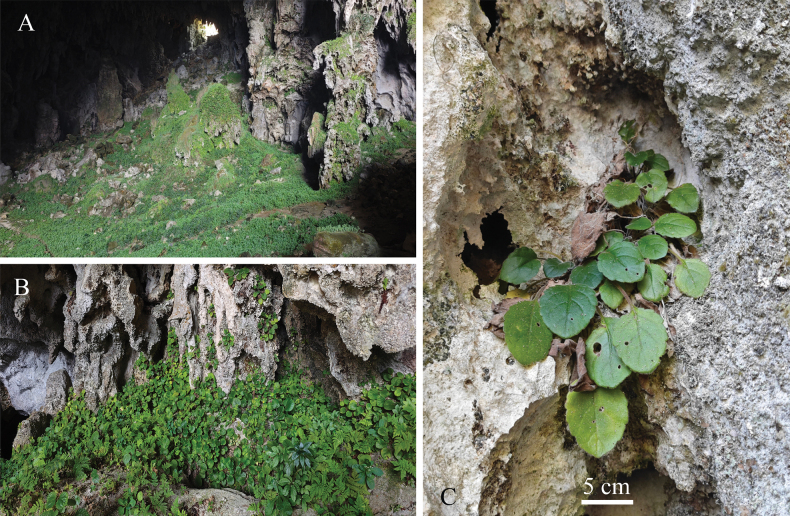
*Petrocodonparviflorus* F.Wen & K.Tan, sp. nov. **A.** Habitat; **B.** Habit; **C.** Plant in the wild (Photographed by Chi Xiong).

##### Type.

China • Guangxi Zhuang Autonomous Region, cultivated in Guilin Botanical Garden, introduced from Hechi City, Fengshan County, Jiangzhou Town, 24°19'N, 106°59'E, 650 m a.s.l., growing on moist shady rock surfaces in a karst cave. 8 August 2024, *Fang Wen* et al. *FZYYZBZ10339*. (***Holotype***: IBK! IBK00470679; ***Isotypes***: IBK!).

**Figure 3.**
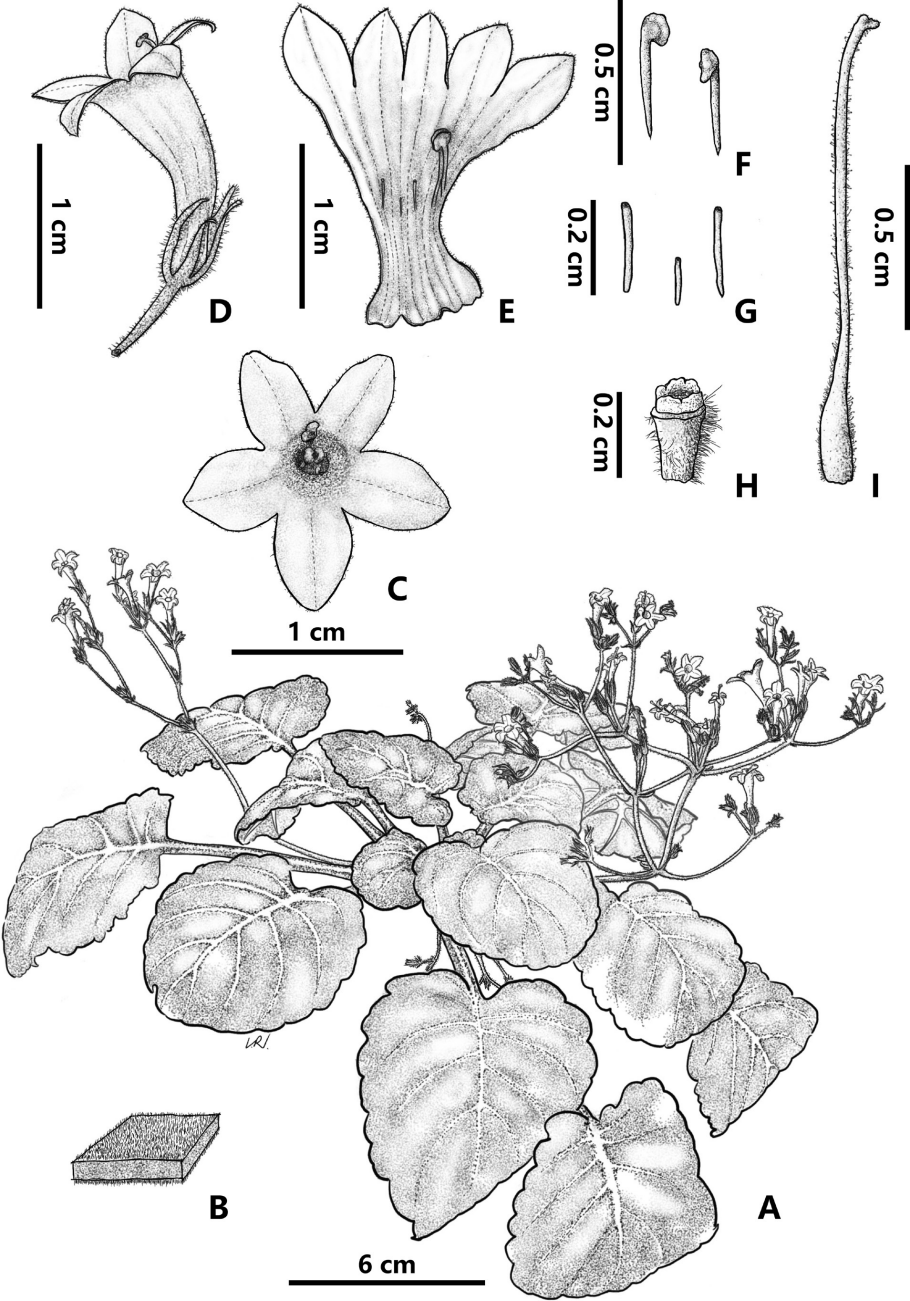
*Petrocodonparviflorus* F.Wen & K.Tan, sp. nov. **A.** Plant in flower; **B.** Leaf surfaces; **C.** Flower, front view; **D.** Flower; **E.** Opened corolla, showing stamens and staminodes; **F.** Stamens; **G.** Staminodes; **H.** Disc; **I.** Pistil (Drawn by Rui-ning Liu).

##### Description.

Perennial herbs. Rhizome brown, subterete, 5–7 cm long, ca. 1 cm in diameter, with distinct internodes, abundant fibrous roots. Leaves 13–19, congested at the apex of rhizome, petiolate; petiole brown, up to ca. 7 cm long, 1.5–4 mm in diameter, transversely elliptic in cross section, slightly grooved adaxially, densely white pubescent; leaf blade slightly chartaceous, green adaxially and pale green abaxially, broadly ovate to suborbicular, 4–6.5 × 4–5.5 cm, apex subacute or obtuse, base cordate to shallowly cordate, oblique, densely white pubescent adaxially and abaxially, margin crenate, lateral veins 4–5 on each side of median vein, impressed adaxially, slightly prominent abaxially. Cymes 10–13, axillary, 3–4-branched, 8–18-flowered; peduncle brownish, 5–9 cm long, 1.5–2 mm in diameter, densely white puberulous pubescent; bracts 2, opposite, green, linear, 5–7 × 1–1.5 mm, outside densely pubescent and inside sparsely pubescent, bracteoles 2, shape similar to bracts but smaller, 3–4 × 0.5–1 mm; pedicels green or brown, 0.5–1 cm long, ca. 1 mm in diameter, puberulous. Calyx 5-partited to the base, lobes equal, narrowly lanceolate, 7–8 × 0.5–1 mm, green to brown, puberulous adaxially, sparsely puberulent abaxially, margin entire, apex acute. Corolla whitish purple to pinkish purple, 1.5–2.5 cm long, 3–5 mm in diameter at the mouth; outside glandular-puberulent, inside puberulent; tube slender, infundibuliform-tubular, 1–1.5 cm long, 1.5–2 mm in diameter at the middle, tube slightly upward curved, 2–2.5 mm in diameter at the base; limb distinctly 2-lipped; adaxial lip 2-lobed to the base, lobes oval, apex acute, 3.5–4 × 2.5–3 mm; abaxial lip 3-lobed to the base, lobes equal, oval, apex acute, 4–4.5 × 3–3.5 mm, with 2 pale yellow nectar guides at the base of the abaxial lip lobe. Stamens 2, adnate to 5–6 mm above the base of the corolla tube, filaments white, 2–2.5 mm long, linear, straight, glabrous; anthers pale yellowish to nearly white, fusiform to ellipsoid, 0.7–1 × 0.6–0.8 mm, glabrous. Staminodes 3, translucent to white, glabrous, lateral ones adnate to 5.5–6 mm above the tube base, 1.5–1.8 mm long, clavate, straight, apex capitate; the central one adnate to 5–5.5 mm above corolla tube base, 1–1.3 mm long. Disc annular, pale yellow, ca. 0.5 mm in height, margin repand. Pistil 1.2–1.4 cm long, ovary pale green, cylindric, 2–2.5 mm long, 0.8–1 mm in diameter, glandular-pubescent; style 10–11.5 mm long, 0.4–0.6 mm in diameter, light green, densely puberulous mixed translucent glandular-puberulous; stigma 2, each ovate, ca. 0.5 mm long, 0.8–1 mm wide, sparsely translucent glandular-hispid. Capsule not seen.

**Figure 4.**
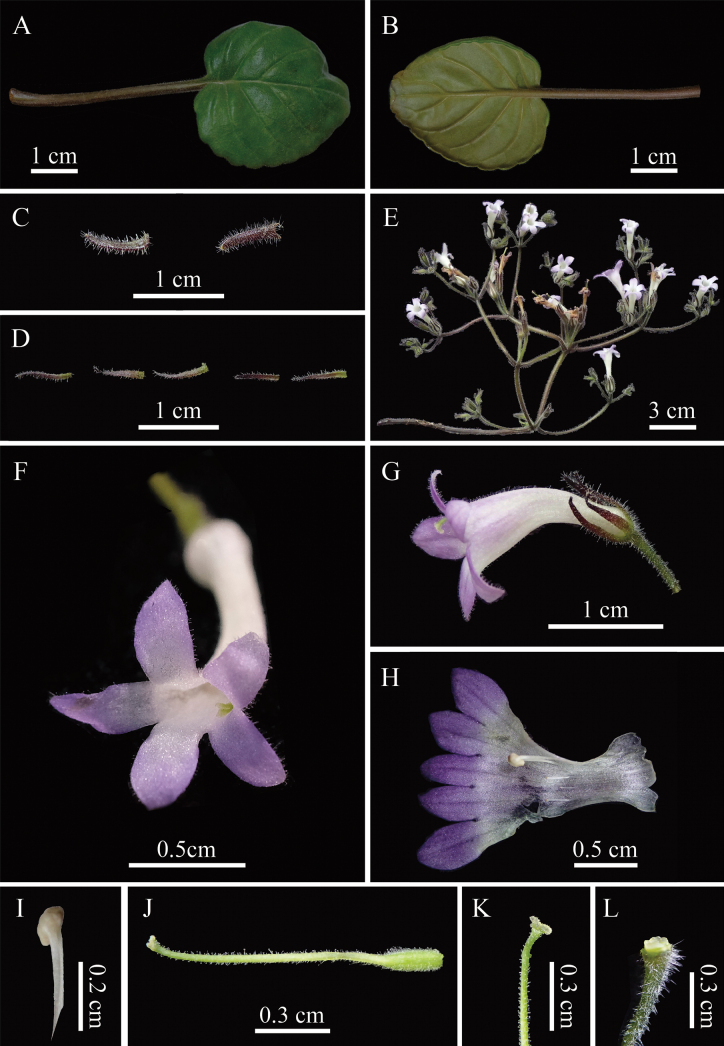
*Petrocodonparviflorus* F.Wen & K.Tan, sp. nov. **A.** Adaxial leaf surface; **B.** Abaxial leaf surface; **C.** Bracts; **D.** Calyx lobes (the first three are adaxial surface and the last two are abaxial surface); **E.** Inflorescence; **F.** Face view of corolla; **G.** Flower in side view; **H.** Opening flower showing stamens and staminode; **I.** Stamens; **J.** Pistil; **K.** Stigma; **L.** Disc (Photographed by Rui-ning Liu).

##### Distribution and habitat.

The new species can only be found at its type locality, Jiangzhou Township, Fengshan County, northwestern Guangxi, China. It grows on moist shady rock surfaces in a karst cave at an altitude of 600–700 m. The average temperature in Fengshan County is 19.7 °C, and the average annual precipitation is 1628.9 mm. The main associated plants of *Petrocodonparviflorus* include *Primulinatribracteata* (W.T.Wang) Mich.Möller & A.Weber, *Mitreolapingtaoi* D.Fang & D.H.Qin, *Elatostemaobscurinerve* W.T.Wang, *Begonialuzhaiensis* T.C.Ku and others.

**Figure 5.**
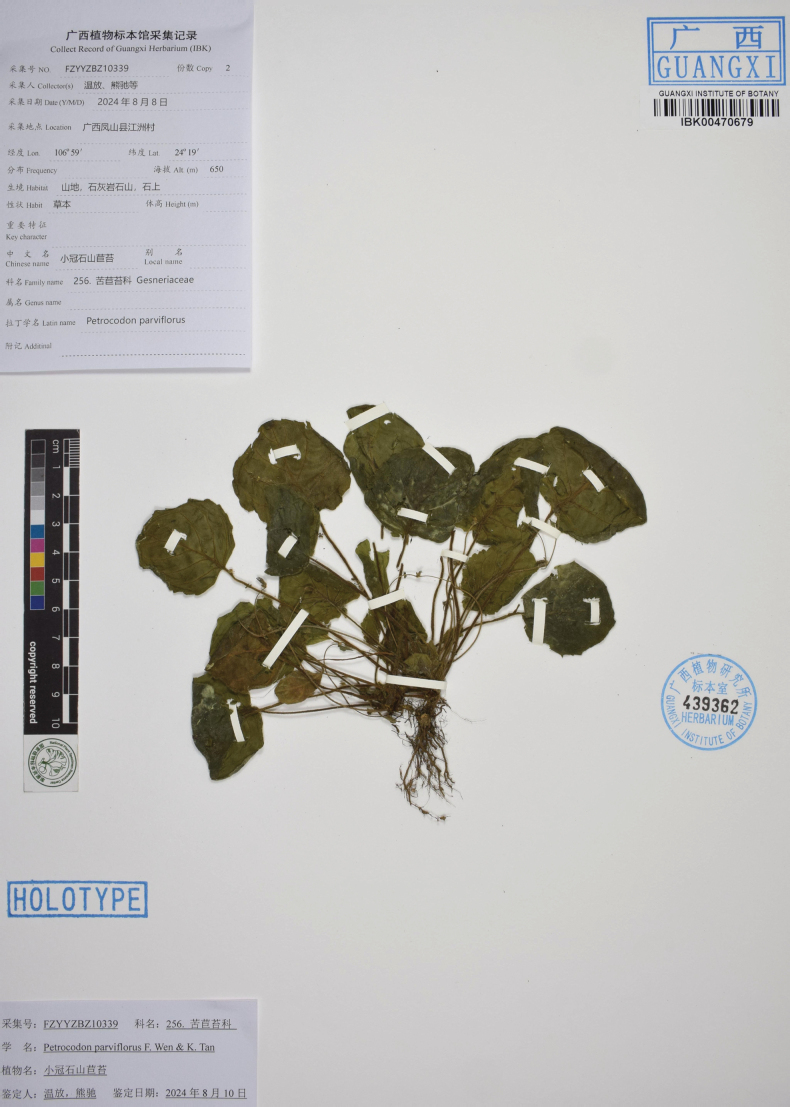
Herbarium type specimens of *Petrocodonparviflorus* F.Wen & K.Tan, sp. nov. (FZYYZBZ10339).

##### Phenology.

Flowering from August to November and fruiting from September to December.

##### Etymology.

The epithet ‘parviflorus’ reflects the relatively small flowers. The corolla is distinctly smaller compared to other species previously classified under the former *Lagarosolen* W.T.Wang, particularly *L.lui* Yan Liu & W.B.Xu (syn. *Petrocodonlui*).

##### Vernacular name.

小冠石山苣苔 (Chinese pronunciation, Xiáo Guàn Shí Shān Jù Tái).

##### Provisional conservation status.

To date, two populations of *Petrocodonparviflorus* have been documented. Both populations grow within karst caves in Jiangzhou Township, Guangxi Zhuang Autonomous Region. The caves essential for the survival of both populations are bisected by a highway. One population is confined to the lower section of a penetration cave, representing a relict subpopulation. Our six years of continuous monitoring (2019–2025) indicate that as of March 2025, this population comprises fewer than 50 mature plants, exhibits limited seedling regeneration, and suffers ongoing habitat degradation driving a persistent population decline. The other population inhabits the entrance complex of a large karst cave, primarily distributed on moist vertical cliff faces within the cave’s entrance zone, with additional occurrences in rock crevices that maintain favorable moisture levels and receive bright scattered light. This population maintains a relatively large size, with more than 6,000 estimated mature individuals and healthy seedling regeneration supporting population sustainability. The cave currently experiences minimal anthropogenic disturbance, primarily limited to cave research activities (e.g., geological and geographical studies), with no observed evidence of significant human impacts elsewhere in the system. The Extent of Occurrence (EOO) for the species is less than 8 km^2^ and the known Area of Occupancy (AOO) is less than 5 m^2^. Consequently, we suggest that the new species *P.parviflorus* should be considered “Vulnerable” [VU, D2] according to current IUCN Red List Categories and Criteria (IUCN 2024).

## ﻿Discussion

The establishment of the genus, *Lagarosolen*, was characterized by an emphasis on three diagnostic features: an elongated corolla tube exceeding twice the limb length, filaments described as linear and straight, and a bilobed style apex with subequal lobes (Wang 1984). Under this historical classification system and the characteristics defined by the aforementioned system, this new species would have been placed with the genus, *Lagarosolen*. However, Weber et al. redefined the genus, *Petrocodon* Hance using molecular data, and *Lagarosolen* was subsequently dissolved and merged into *Petrocodon* (Weber et al. 2011). This expansion resulted in a morphologically diverse *Petrocodon*, making it challenging to determine a member according to common and stable diagnostic traits for the genus.

Accordingly, our phylogenetic topology (Fig. 1) aligns with previous phylogenetic analyses of *Petrocodon* (Zhang et al. 2023; Shi et al. 2025). And our analyses of molecular data show that *Petrocodonparviflorus* falls into a highly supported clade. Based on both morphological characteristics and molecular evidence, we can confirm that the new species found at Fengshan County belongs to the redefined *Petrocodon*. Moreover, the new species is characterized by its notably smaller flowers and overall size within *Petrocodon*, so it is named “parviflorus”, meaning “small corolla”. The variations in these morphology between the two closely related species can be seen in Table 2.

**Table 2.** Comparison of *Petrocodonparviflorus* sp. nov. and *P.lui*.

Character	*Petrocodonparviflorus*	*P.lui*
Leave	13–19, densely white pubescent adaxially and abaxially	6–10, adaxially glabrous or sparsely puberulent, abaxially puberulent
Cymes	10–13, 3–4-branched	3–5, 1–2-branched
Bract	5–7 × 1–1.5 mm	3–4 × 0.3–0.4 mm
Calyx	lobes puberulous adaxially, sparsely puberulent abaxially	lobes puberulent adaxially, glabrous abaxially.
Corolla	adaxial lip lobes oval, apex acute, 3.5–4 × 2.5–3 mm; abaxial lip 3-lobed to the base, lobes oval, apex acute, 4–4.5 × 3–3.5 mm	adaxial lip lobes oblong, obovate to suborbicular, apex round, 6–6.5 × 5–5.5 mm; abaxial lip 3-lobed to or over the middle, lobes oblong, obovate to suborbicular, apex round, 7–7.5 × 5–5.5 mm
Stamens	adnate to 5–6 mm above the corolla base, filaments glabrous	adnate to 8–10 mm above the corolla base, filaments sparsely glandular
Staminode	the central staminode one 1–1.3 mm long	the central staminode one 0.5–0.8 mm long
Pistil	ovary 2–2.5 mm long,0.8–1 mm in diameter; style 10–11.5 mm long	ovary 3–3.5 mm long,1.5–1.8 mm in diameter; style 4–6 mm long

## Supplementary Material

XML Treatment for Petrocodon parviflorus

## ﻿Additional information

### Use of AI

No use of AI was reported.

### Author ORCIDs

Rui-Ning Liu https://orcid.org/0009-0004-7171-2221

Ke Tan https://orcid.org/0000-0002-9036-163X

Chi Xiong https://orcid.org/0000-0002-9082-2539

Song-Tao He https://orcid.org/0009-0005-5223-7522

Fang Wen https://orcid.org/0000-0002-3889-8835

### Data availability

All of the data that support the findings of this study are available in the main text.
